# Supplementary material for: Selective COVID-19 Coinfections in Diabetic Patients with Concomitant Cardiovascular Comorbidities Are Associated with Increased Mortality
Source: Pathogens. 2022 Apr 25;11(5):508. doi: 10.3390/pathogens11050508 (PMC9145452; doi:10.3390/pathogens11050508)
Supplement: Supplementary file 1 [file pathogens-11-00508-s001.zip › pathogens-1662546-supplementary.pdf]

Supplementary Table S1. Influence of bacterial coinfections, diabetes, and related heart comorbidities in mortality rates of COVID-19 patients

	bacteria	**Asthma**	**HTN**	**Diabetes**	**CKD**	**CLD**	**HF**	**IHD**	**Death**
1	*K.pneumoniae*	yes	yes	yes	no	no	no	yes	yes
2	*K.pneumoniae*	no	yes	yes	no	no	no	yes	yes
3	*K.pneumoniae*	no	yes	yes	no	no	no	yes	yes
4	*K.pneumoniae*	no	yes	yes	no	no	no	yes	yes
5	*K.pneumoniae*	no	yes	no	no	no	no	yes	yes
6	*K.pneumoniae*	no	yes	yes	no	no	no	yes	yes
7	*K.pneumoniae*	yes	yes	yes	no	yes	yes	yes	yes
10	*K.pneumoniae*	yes	yes	yes	no	yes	yes	yes	yes
11	*K.pneumoniae*	yes	yes	yes	yes	yes	no	yes	yes
12	*K.pneumoniae*	yes	yes	yes	yes	yes	no	yes	yes
13	*K.pneumoniae*	no	yes	yes	no	no	no	no	yes
23	*K.pneumoniae*	no	yes	yes	yes	no	no	yes	yes
37	*K.pneumoniae*	yes	yes	yes	no	yes	no	yes	yes
45	*K.pneumoniae*	yes	yes	yes	no	no	no	yes	yes
50	*A.baumannii*	no	yes	yes	yes	no	no	yes	yes
51	*A.baumannii*	yes	yes	yes	yes	no	no	yes	yes
52	*A.baumannii*	yes	yes	yes	yes	yes	no	yes	yes
53	*A.baumannii*	yes	no	yes	no	no	no	yes	yes
56	*A.baumannii*	yes	yes	no	yes	no	no	no	yes
62	*A.baumannii*	yes	yes	yes	no	no	no	no	yes
63	*A.baumannii*								yes
65	*A.baumannii*	no	yes	yes	yes	no	no	no	yes
69	*A.baumannii*	no	yes	yes	yes	yes	no	yes	yes
70	*A.baumannii*								yes
71	*A. baumannii*	yes	yes	yes	no	yes	no	yes	yes

84	*E.coli*	no	yes	yes	no	no	no	no	yes
86	*E.coli*	no	yes	yes	yes	no	no	no	yes
106	*E.coli*	yes	yes	yes	no	yes	no	yes	yes
116	*P.aeruginosa*	yes	no	yes	yes	yes	no	yes	yes
123	*Serratia*			yes					yes
124	*Serratia*	no	yes	yes	no	yes	no	yes	yes
125	*morganella*	yes	yes	yes	no	no	no	yes	yes
126	*morganella*	no	yes	yes	yes	yes	no	yes	yes
129	*C. koseri*	yes	yes	yes	yes	no	yes	yes	yes
